# Combining chemical genomics screens in yeast to reveal spectrum of effects of chemical inhibition of sphingolipid biosynthesis

**DOI:** 10.1186/1471-2180-9-9

**Published:** 2009-01-14

**Authors:** Danielle Kemmer, Lianne M McHardy, Shawn Hoon, Delphine Rebérioux, Guri Giaever, Corey Nislow, Calvin D Roskelley, Michel Roberge

**Affiliations:** 1 Department of Biochemistry and Molecular Biology, University of British Columbia, Vancouver, Canada; 2 Stanford Genome Technology Center, Stanford University, Palo Alto, CA, USA; 3 Department of Pharmaceutical Sciences, University of Toronto, Toronto, Canada; 4 Donnelly Centre for Cellular and Biomolecular Research, University of Toronto, Toronto, Canada; 5 Department of Molecular Genetics, University of Toronto, Toronto, Canada; 6 Banting and Best Department of Medical Research, University of Toronto, Toronto, Canada; 7 Department of Cellular and Physiological Sciences, University of British Columbia, Vancouver, Canada

## Abstract

**Background:**

Single genome-wide screens for the effect of altered gene dosage on drug sensitivity in the model organism *Saccharomyces cerevisiae *provide only a partial picture of the mechanism of action of a drug.

**Results:**

Using the example of the tumor cell invasion inhibitor dihydromotuporamine C, we show that a more complete picture of drug action can be obtained by combining different chemical genomics approaches – analysis of the sensitivity of *ρ*^0 ^cells lacking mitochondrial DNA, drug-induced haploinsufficiency, suppression of drug sensitivity by gene overexpression and chemical-genetic synthetic lethality screening using strains deleted of nonessential genes. Killing of yeast by this chemical requires a functional mitochondrial electron-transport chain and cytochrome c heme lyase function. However, we find that it does not require genes associated with programmed cell death in yeast. The chemical also inhibits endocytosis and intracellular vesicle trafficking and interferes with vacuolar acidification in yeast and in human cancer cells. These effects can all be ascribed to inhibition of sphingolipid biosynthesis by dihydromotuporamine C.

**Conclusion:**

Despite their similar conceptual basis, namely altering drug sensitivity by modifying gene dosage, each of the screening approaches provided a distinct set of information that, when integrated, revealed a more complete picture of the mechanism of action of a drug on cells.

## Background

Knowledge of the different proteins and cellular processes affected by chemicals is necessary to rationally guide drug discovery and development. This is a difficult challenge because unbiased techniques to sample all possible target proteins and pathways are currently lacking. The observation that modifying the amount or activity of a gene product via mutation, overexpression, downregulation or deletion can change the response of a cell to a chemical [1,2] raises hope that systematic genome-wide screens of drug sensitivity can help uncover direct and indirect drug targets as well as modifiers of cellular responses to chemicals.

The yeast *Saccharomyces cerevisiae *is currently the eukaryotic model organism with the most comprehensive genome-wide collections of mutant strains available to monitor drug sensitivity. *S. cerevisiae *exists as a haploid or as a diploid. Deleting 1 of the 2 copies of a gene in diploid strains can reduce its expression, and a set of ~6,000 heterozygous diploid strains covering nearly all essential and nonessential genes is available. Complete deletion of nonessential genes eliminates their expression and sets of ~4,900 haploid and homozygous diploid deletion mutants are also available. *S. cerevisiae *can be easily transformed and increased gene expression can be achieved by introducing plasmids containing genomic DNA fragments or gene-coding regions controlled by inducible promoters [3]. The unicellular nature of yeast and its ability to grow on liquid or solid media also make it amenable to high-throughput drug studies.

A number of studies have shown that reducing the copy number of essential or nonessential genes from 2 to 1 in diploid cells may increase the sensitivity of the cell to a drug (termed drug-induced haploinsufficiency) and can point to candidate target genes [4-6]. Haploid or homozygous diploid deletion collections contain only deletions of nonessential genes. Screening these collections for hypersensitivity to a small molecule reveals genes that buffer the drug target pathway, not the direct drug targets and comparison of the profile of chemical-genetic synthetic lethality with a compendium of chemical-genetic or genetic interaction profiles can aid in deciphering its targets [7,8]. Increased gene expression can lead to suppression of drug sensitivity and also reveal target genes [3,9]. Studies of the mechanism of action of drugs using genome-wide approaches in yeast have tended to focus on 1 of these 3 approaches [3,5,8]. While each generally reveals important clues, they draw only a partial picture of the mechanism of action of chemicals. For example, a drug-induced haploinsufficiency screen of the cancer cell invasion inhibitor dihydromotuporamine C (dhMotC) showed that the compound targets sphingolipid biosynthesis and affects the actin cytoskeleton [6], but did not reveal whether other cellular functions were affected and gave no indication of cell death mechanisms involved.

Genome-wide studies of drug mechanism of action have mainly concentrated on nuclear-encoded genes. Genes encoded by mitochondrial DNA, which include components of the mitochondrial translational machinery and 8 mitochondrial proteins, have not received as much attention. Yet mitochondria are recognized as important regulators of cell death in addition to their central role in energy production [10]. Although yeast displays only some of the characteristics of apoptosis described in humans, many cellular features of the cell death pathway in mammalian cells have been identified in yeast [11]. Therefore, it may also be important to examine the contribution of the mitochondrial genome to drug action.

Here we first examine the importance of the mitochondrial genome to drug sensitivity using *ρ*^0 ^*petite *strains deleted of mitochondrial DNA. We then examine the value to elucidating the mechanism of action of dhMotC of combining screening of *ρ*^0 ^cells with 3 genome-wide screening approaches: drug-induced haploinsufficiency, chemical-genetic synthetic lethality and suppression of drug sensitivity by increased gene expression. We find that despite their similar conceptual basis, namely altering drug sensitivity by modifying gene dosage, the 3 approaches can provide distinct sets of information that, when integrated, reveal a much more complete picture of the spectrum of effects of a drug on cells.

## Results and discussion

### Screen for mitochondria-dependent inhibitors of yeast growth

Halo assays, traditionally used in antibacterial screens, can be used to assess cytotoxic properties of chemicals in yeast [12]. Fungistatic and fungicidal chemicals spotted onto plates containing a lawn of *S. cerevisiae *growing in soft agar cause zones of growth inhibition (halos) that are easily detected by visual inspection. Robotic pinning enables high-density arraying of compounds for increased throughput.

We used the halo assay to screen approximately 3,500 FDA-approved drugs and bioactive chemicals [13] as well as in-house chemicals for inhibition of yeast growth. Chemicals were pin-transferred onto agar containing the wild type yeast strain BY4741 [14] or strain FY1679-28C/TDEC [15] with deletion of 2 transcription factors, *PDR1 *and *PDR3*, that regulate a wide range of multidrug resistance genes, to increase the likelihood of identifying active compounds. To determine the effect of functional mitochondria on drug sensitivity, the screen was also carried out on respiratory-deficient *ρ*^0 ^*petite *mutants of the 2 strains. The strains lacking functional mitochondria were generated by propagating cells in the presence of ethidium bromide, resulting in the selective loss of the mitochondrial genome, including several essential components of the electron-transport chain, which renders cells respiratory-deficient [16]. The *ρ*^0 ^*petite *strains were unable to grow on glycerol, a nonfermentable carbon source, confirming their inability to generate ATP by mitochondrial oxidative phosphorylation (data not shown).

**Figure 1:**
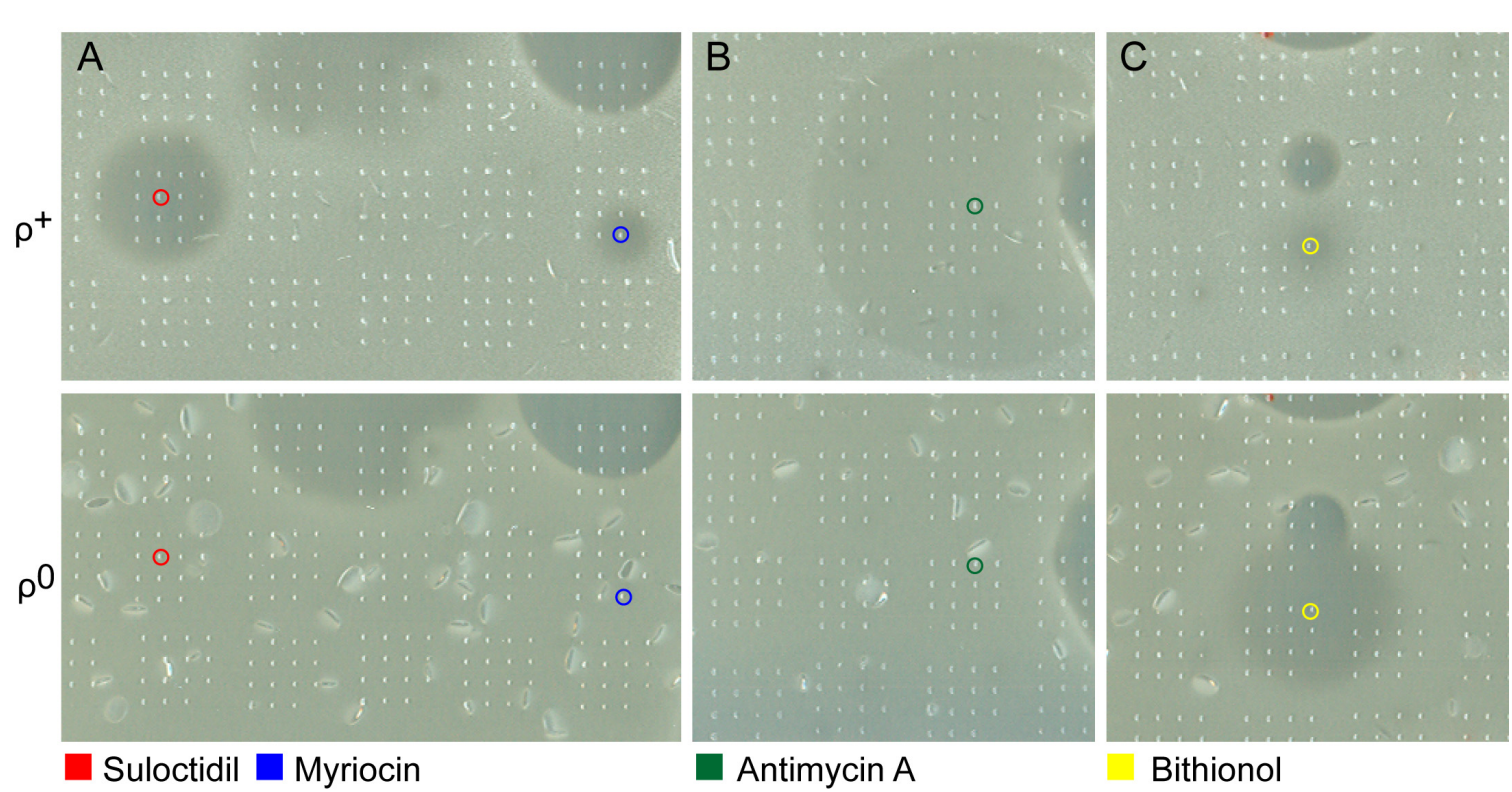
**Halo screen of chemical collection with *ρ*^+ ^and *ρ*^0 ^FY1679-28C/TDEC**. (A) Suloctidil and myriocin selectively inhibit growth of *ρ*^+ ^cells; (B) Antimycin A selectively slows growth of *ρ*^+^; (C) Increased sensitivity of *ρ*^0 ^cells to bithionol.

Plates were inspected after 48 h incubation at 30°C and halos > 2 mm in diameter were scored. 51 chemicals inhibiting the growth of FY1679-28C/TDEC were identified (Table 1), 39 of which also inhibited the growth of BY4741. Only 4 chemicals affected the growth of wild type and *ρ*^0 ^cells differently. Suloctidil, myriocin, dhMotC and antimycin A inhibited respiratory-competent strains but failed to inhibit the growth of the *ρ*^0 ^strains (Figure 1A and 1B). Suloctidil, myriocin and dhMotC caused the appearance of clear halos that did not contain observable growth, characterizing these compounds as fast-acting antifungals, while antimycin A enabled slow growth within the area of the halo (Figure 1B). A single chemical, identified as bithionol, strongly inhibited the growth of the *ρ*^0 ^but inhibited the growth of wild type yeast very weakly (Figure 1C). Since antimycin A directly blocks the mitochondrial electron-transfer chain, a difference in drug response between wild type and *ρ*^0 ^strains was to be expected and this compound was not studied further.

**Table 1:** 51 growth-inhibitory compounds identified in halo toxicity screen

Compound	*ρ*^+^	*ρ*^0^
Antibiotics and antiseptics		
**Antimycin A**	**++**	**-**
Cefoperazone sodium	++	++
Cetylpyridinium chloride	+	+
Chloroxine	+	+
Hexachlorophene	++	++
**Myriocin**	**++**	**-**
Thimerosal	++	++
Tunicamycin B	++	++
Anticancer		
Swainsonine	++	++
Dequalinium analog C-14 linker	+	+
**dhMotC**	**++**	**-**
Azoles		
Bifonazole	++	++
Butoconazole nitrate	+++	+++
Clotrimazole	+++	+++
Econazole nitrate	+++	+++
Enilconazole	++	++
Isoconazole	+++	+++
Ketoconazole	++	++
Miconazole	+++	+++
Miconazole nitrate	+++	+++
Oxiconazole nitrate	+++	+++
Sertaconazole nitrate	+++	+++
Sulconazole nitrate	+++	+++
Detergents		
Cetrimonium bromide	+	+
Digitonin	+	+
Flavonoids		
Luteolin	++	++
Other		
Adamantamine fumarate	++	++
Amiodarone hydrochloride	+	+
Anthothecol	+	+
Benzalkonium chloride	+	+
**Bithionol**	**+**	**+++**
Cedrelone	+	+
Celastrol	+	+
Dequalinium dichloride	++	++
Elaidylphosphocholine	+	+
Ellagic acid	+	+
Gentian violet	++	++
Miltefosine	+	+
Obtusaquinone	+	+
Phenylmercuric acetate	+++	+++
Phytosphingosine	+	+
Plumbagin	+	+
Pyrithione zinc	++	++
Pyrvinium pamoate	+	+
Rapamycin	+++	+++
Shikonin	+	+
SKF96365	++	++
**Suloctidil**	**++**	**-**
Thiram	+	+
Tomatidine hydrochloride	+	+
Totarol	+	+

Comparison between respiratory-proficient and -deficient yeast strains.

The results of the halo screen were confirmed and extended using a quantitative liquid growth assay. Suloctidil (50 μM), myriocin (0.25 μM) and dhMotC (60 μM) all inhibited the growth of the wild type strain while *ρ*^0 ^cells were resistant (Table 2). *P*^0 ^strains have previously been shown to have increased expression of multidrug resistance genes [17], which could have explained their increased resistance to the 4 chemicals. However, this is not the case since increased resistance was also observed in the *ρ*^0 ^strain lacking *PDR1 *and *PDR3*, that cannot express multidrug resistance genes, in agar (Table 1), as well as in liquid assay (data not shown). Therefore, resistance to the growth inhibitory effect of the chemicals is due to the lack of mitochondrial function.

**Table 2:** Liquid growth assay for drug sensitivity of *ρ*^+ ^and *ρ*^0 ^strains

Compound	Growth (% of drug-free assay)	
	*ρ*^+^	*ρ*^0^
Suloctidil (50 μM)	43	98
Myriocin (250 nM)	23	93
dhMotC (60 μM)	47	100

Myriocin binds to and inhibits serine palmitoyltransferase, responsible for the first committed step of *de novo *synthesis of sphingosine, ceramide and complex sphingolipids [18]. DhMotC, an inhibitor of tumor cell invasion [19], also inhibits sphingolipid biosynthesis and genes of the sphingolipid biosynthesis pathway show dhMotC-induced haploinsufficiency [6]. Interestingly, suloctidil was recently shown to inhibit acid sphingomyelinase, a lysosomal enzyme catalyzing the degradation of sphingolipids and generating ceramide, which can be metabolised into sphingosine [20].

These results show that the majority of chemicals that inhibit yeast growth do not require functional mitochondria to exert their effect but that 3 compounds affecting sphingolipid metabolism all require functional mitochondria to inhibit growth. We then further explored the requirement for functional mitochondria in the mechanism of action of 1 of these chemicals, dhMotC, using genetic screens and biological assays.

### Prolonged exposure to dhMotC kills yeast

**Figure 2:**
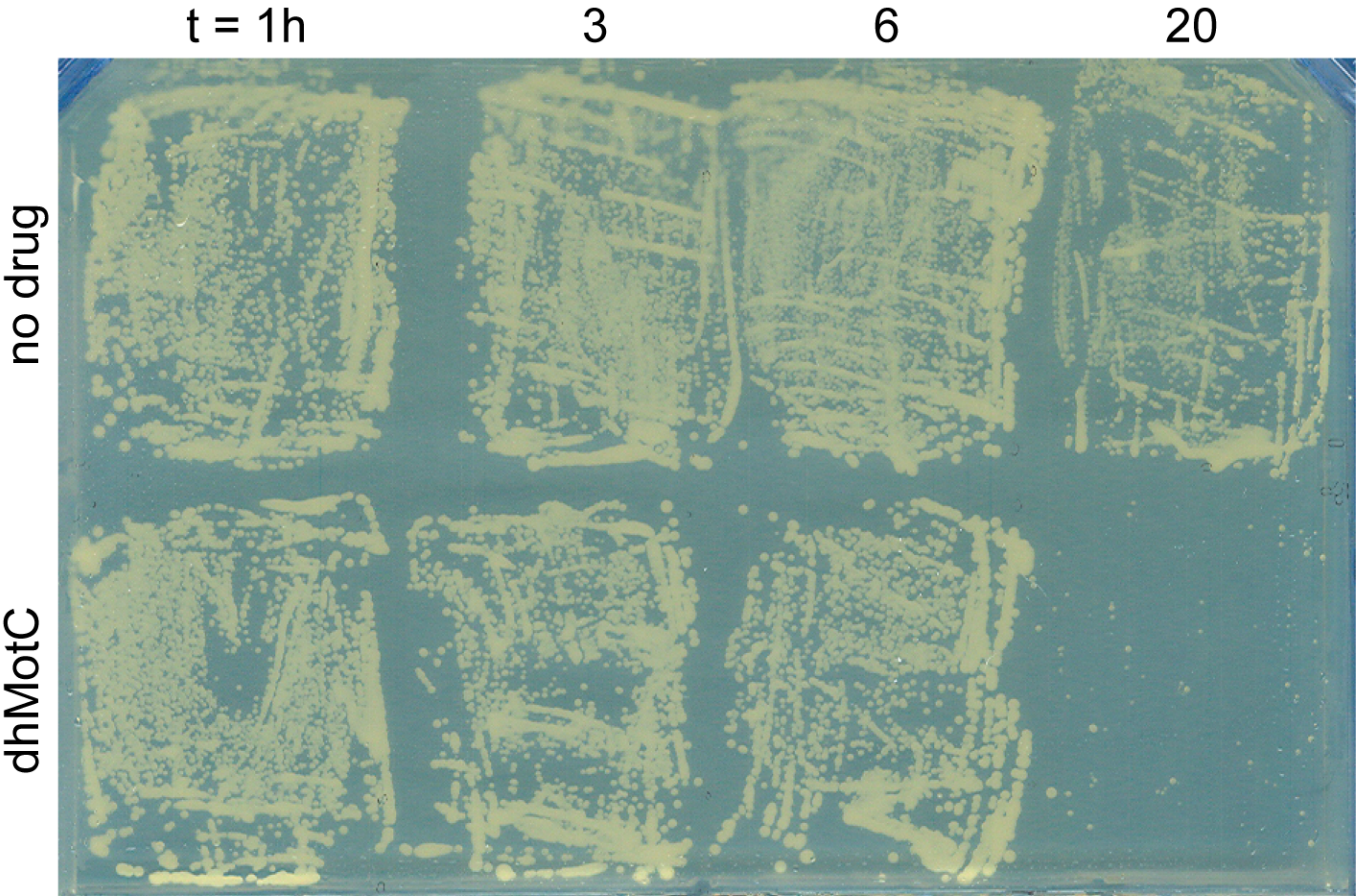
**Viability test of FY1679-28C/TDEC yeast strain exposed to dhMotC**. Short exposure times result in reversible growth inhibition. There is no observable growth after long drug exposure.

Growth-inhibitory compounds can reversibly prevent cell proliferation (cytostatic activity) or induce death (cytocidal activity). To distinguish between these outcomes, cells were exposed to inhibitory concentrations of dhMotC in liquid culture for different times and equal cell numbers were plated onto drug-free agar plates for 2 days at 30°C. Cells exposed to dhMotC for 1, 3 or 6 hours all formed the same number of colonies as untreated cultures. However, exposure to dhMotC for 20 h resulted in no colony growth (Figure 2). These observations show that dhMotC exposure initially triggers a reversible growth arrest that eventually leads to cell death after longer exposure.

### DhMotC sensitivity suppressor screen reveals genes involved in mitochondrial function

Screens using increased gene dosage, relying on the assumption that increased levels of a protein targeted by a drug increase resistance to the drug, can help identify specific drug targets [9]. Drug sensitivity suppressor screens can be carried out with pooled genomic library transformants, leading to enrichment of resistant strains and depletion of hypersensitive strains, relative to untreated pools. Analysis of relative strain sensitivity is performed by hybridization of labelled DNA to an oligonucleotide tag array [21].

**Figure 3:**
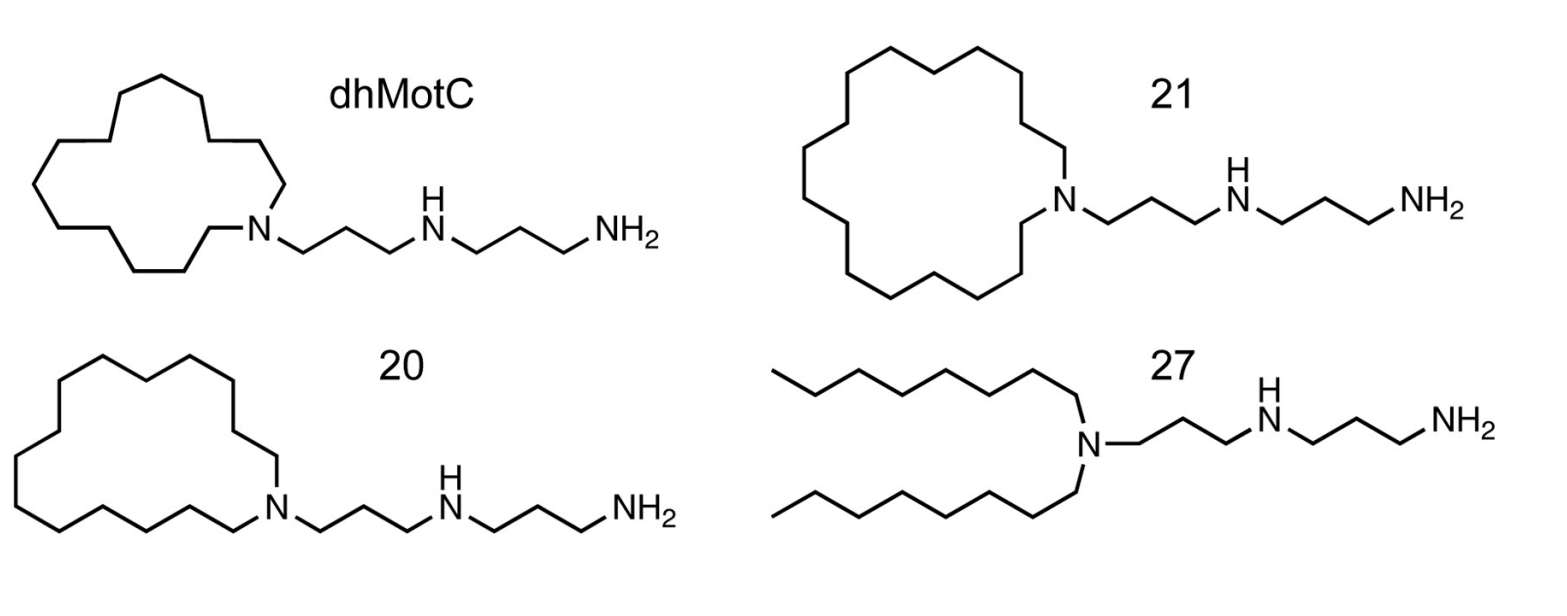
**Structural formulae of dhMotC and close analogues**.

A pooled collection of yeast strains expressing genes from a random genomic DNA fragment library was exposed to dhMotC and resistant strains were identified. Similar experiments were carried out using 3 close structural analogues (Figure 3). Syntenic regions (i.e. genomic DNA fragments) containing genes linked from the same locus that scored highly for dhMotC and at least 1 analogue, compared to an untreated pool of strains, are shown in Table 3. All genomic DNA fragments conferring increased resistance contained more than 1 gene. To identify individual genes conferring resistance, the highest-scoring region for the 2 most potent invasion inhibitors, dhMotC and analogue 20, linking genes *AVO1 *and *ATP19*, was selected, as was the only syntenic region common to all analogues tested, linking genes *SDS22 *and *ACP1*. Each gene was overexpressed individually and its effect on yeast growth in the presence of 30 μM dhMotC was determined. The overexpression of *ATP19 *(log_10 _= 0.0142) and *ACP1 *(log_10 _= 0.0137) conferred a 10-fold and 7-fold growth increase compared to *AVO1 *(log_10 _= 0.0014) and *SDS22 *(log_10 _= 0.0019) respectively, revealing the genes encoding mitochondrial proteins from each syntenic region as the suppressors of growth inhibition.

**Table 3:** Dosage suppressor screen

Linked genes\Analogue	dhMotC	20	21	27
	Average log2 fold ratio treated vs. control			
*ARO8 MCM6*	3.12			3.41
*AVO1 ATP19*	4.13	2.37		
*GAA1 ALT1*	2.13			2.41
*HYS2 SUI2 YJR008W*	2.20			2.43
*BFR1 MRM1 HIS3*	2.01		2.32	2.43
*MNN11 YJL181W ATP12 PFD1*	1.71			1.98
*MTF2 PRP11 SIR2*	2.04			1.72
*NST1 RHO2*	2.03			3.02
*SDS22 ACP1*	3.09	1.71	1.95	2.60
*SPO1 YNL011C YNL010W IDP3 ASI3*	3.88		2.15	3.11
*YHR162W SOL3 DNA2*	2.92			2.99
*YML081W DUS1 YML079W CPR3*	1.75			2.81
*EBS1 UME6 MSS4 YDR210W*	2.35			2.43

Syntenic regions enriched after treatment with motuporamines.

Atp19p is a subunit of the mitochondrial F_0_F_1 _ATP synthase, a large enzyme complex involved in ATP synthesis. This peripheral membrane protein has been proposed to be involved in the arrangement of the ATP synthase dimer but it is not required for the formation of enzymatically active ATP synthase and its precise role remains unclear [22]. Acp1p is a mitochondrial matrix acyl carrier protein that is involved in fatty acid biosynthesis [23] and its deletion causes a respiratory-deficient phenotype. Acp1p is believed to be involved in the biosynthesis of octanoate, a precursor to lipoic acid. Analysis of the genes shown in Table 3 for biological processes showed an enrichment in genes linked to mitochondrial function (*ATP19*, *ALT1*, *MRM1*, *ATP12*, *MTF2*, *ACP1*, *IDP3*, *YHR162W*, *CPR3*), spanning a wide variety of mitochondrial processes including ATP synthase complex assembly, rRNA and mRNA modification and translation, protein folding, NADPH generation, metabolic processes such as fatty acid beta oxidation and isocitrate metabolism, as well as genome maintenance. Overall, these results indicate that increased mitochondrial function reduces sensitivity to dhMotC.

**Figure 4:**
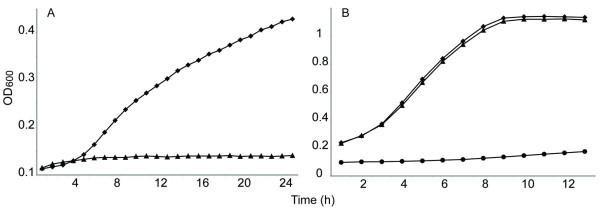
**Hypersensitivity of cells grown on nonfermentable glycerol to dhMotC**. Growth of respiratory-proficient or -deficient yeast (OD_600_) as function of time in hours in liquid culture under different conditions: Growth in the presence of DMSO (Black diamond) or dhMotC (Black triangle). (A) *P*^+ ^strain in glycerol with 5 μM dhMotC; (B) *P*^0 ^strain in glucose with 100 μM dhMotC. Lack of growth of the *ρ*^0 ^strain in glycerol (Black circle).

To further examine the link between dhMotC sensitivity and mitochondrial function, cells were forced to rely exclusively on mitochondria for ATP production by growing them in glycerol, a nonfermentable carbon source. If mitochondrial function is required for dhMotC to kill yeast, then growth in these conditions should render yeast hypersensitive to the drug. Indeed, yeast grown in glycerol as the sole carbon source were highly sensitive to 5 μM dhMotC, a concentration that is sub-inhibitory in medium containing glucose (Figure 4A). *P*^0 ^cells lacking functional mitochondria were completely resistant even to 100 μM dhMotC (Figure 4B). Because functional mitochondria are not essential for yeast cell survival (*ρ*^0 ^strains are viable), these results indicate that dhMotC triggers a mitochondria-dependent cell death mechanism.

### Cell death requires cytochrome c heme lyase

Mitochondria have been implicated in programmed cell death mechanisms in yeast [10]. We next tested a set of mutants of core players in the mitochondria-dependent death response for their sensitivity to dhMotC. We included *aif1Δ *and *mca1Δ*, which are both mutants of important mitochondrial cell death effectors, and *cyc3Δ *and the double mutant *cyc1Δcyc7Δ *[24] which lack mature cytochrome c.

**Figure 5:**
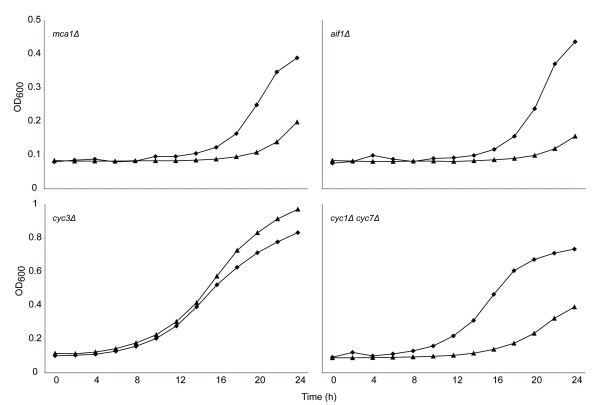
**dhMotC sensitivity of haploid strains deleted of cell death-related genes**. Growth of mutants (OD_600_) as function of time in hours in YPD liquid culture under 2 different conditions: no drug control DMSO (Black diamond) and 100 μM dhMotC (Black triangle).

Mutants were exposed to 100 μM dhMotC for 24 h and growth was compared to untreated controls. *Cyc3Δ *was resistant to the compound while *aifΔ*, *mca1Δ *and *cyc1Δcyc7Δ *were strongly inhibited at this high concentration of dhMotC (Figure 5). *CYC3 *encodes cytochrome c heme lyase, an enzyme catalyzing covalent attachment of the heme group to apocytochrome c [25]. While *S. cerevisiae *possesses 2 forms of cytochrome c, encoded by *CYC1 *and *CYC7 *respectively, *cyc3Δ *mutants lack both holocytochromes c. Heme lyase deficiency also prevents mitochondrial import of the apocytochromes [26].

Overexpression of mammalian Bcl-2 can protect from apoptosis-related death mechanisms in yeast, resulting in cell survival [27]. To test whether cells treated with dhMotC could be rescued by Bcl-2, we overexpressed human Bcl-2 in yeast cells exposed to the compound. Human Bcl-2 was unable to rescue drug-exposed cells and yeast sensitivity to dhMotC was similar to cells without Bcl-2 (data not shown).

Based on our observations that *aif1Δ*, *mca1Δ *and *cyc1Δcyc7Δ *strains were sensitive to dhMotC and that drug-induced cell death could not be rescued by mammalian Bcl-2, we assume that these apoptosis-related genes are not directly involved in the death mechanism triggered by dhMotC. However, since *cyc3Δ *was entirely resistant to the drug, cytochrome c heme lyase plays a specific role in the underlying mitochondrial pathway of cell death.

### Chemical-genetic synthetic lethality screen reveals effects of dhMotC on vacuolar pH and vesicle-mediated transport

To further characterize the cellular effects of dhMotC, we conducted a chemical-genetic synthetic lethality screen using the *S. cerevisiae *haploid deletion set. In principle, synthetic lethality describes genetic interactions in which the combination of 2 nonlethal mutations results in lethality. The method has been applied to identify cellular pathways that "buffer" each other biologically to help decipher gene function(s) of individual pathway members [28]. Global synthetic lethality analysis between null alleles provides a means to identify genes required for redundant biological processes or functioning in parallel pathways. In the same way, testing viable mutants for hypersensitivity to a chemical compound reveals chemical genetic interactions that consist of a set of genes that buffer the cell from defects in drug target activity and identifies specific biological processes that are intricately involved, but are not directly targeted by the drug [7].

**Figure 6:**
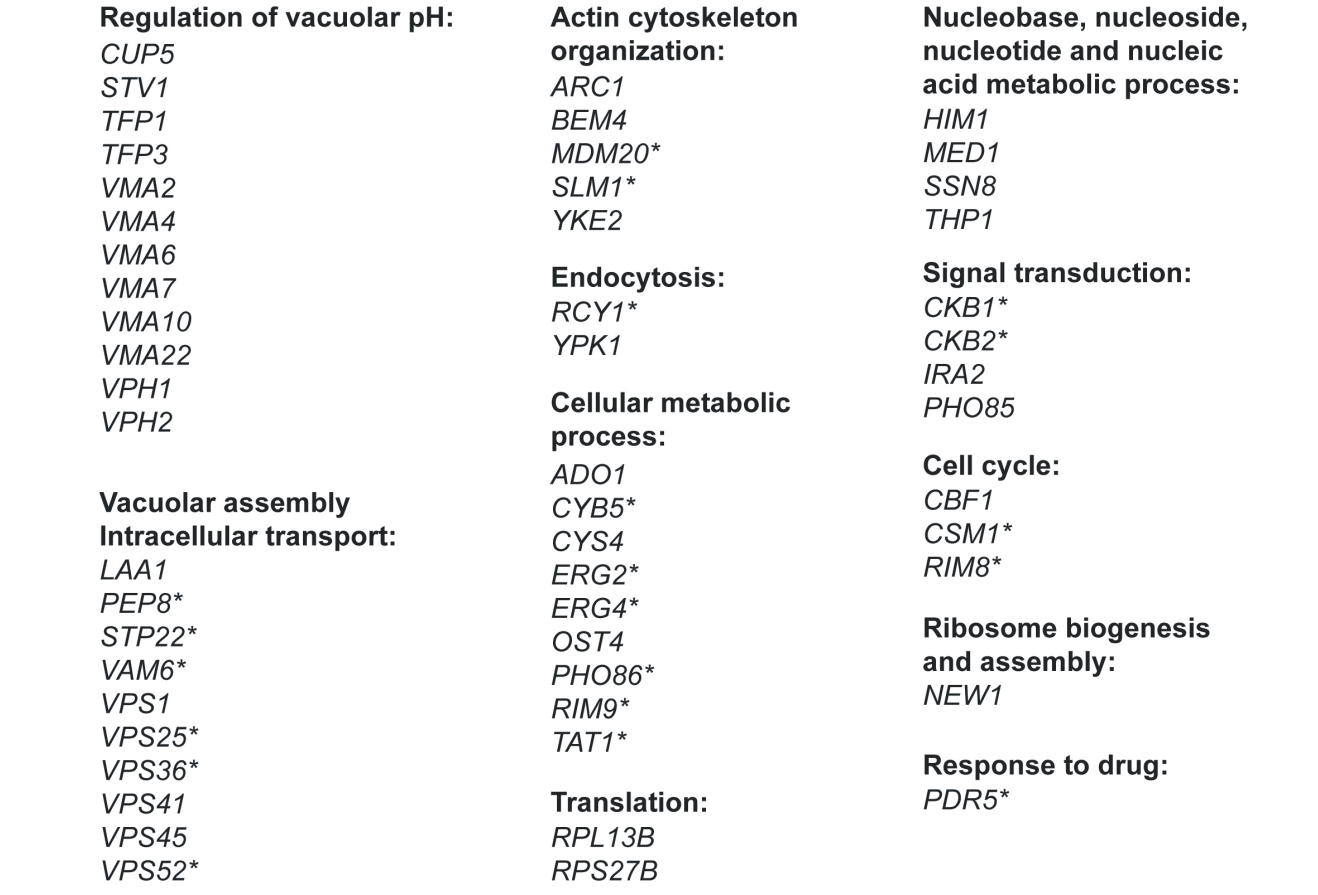
**53 nonessential genes synthetic lethal with dhMotC**. *: MDR genes as defined in Hillenmeyer *et al*. [30].

We screened ~4,700 viable yeast deletion mutants for hypersensitivity to dhMotC by arraying strains onto agar plates containing a sublethal concentration of dhMotC and scoring reduced colony formation. The plates were incubated at 30°C and colony growth was compared over a period of 4 days. Each mutant was arrayed in duplicate and the screen was carried out twice. Strains displaying increased sensitivity to dhMotC in both screens are shown in Figure 6. The list of sensitive strains includes 53 nonessential genes implicated in a variety of biological processes. We found that over 40% of these 53 mutants (22 genes, see Figure 6, first column) were either components of the vacuolar H^+^-ATPase (V-ATPase) required for the activity of the proton pump [29], or were implicated in vacuolar assembly and vesicle-based intracellular transport.

A recent chemical-genetic synthetic lethality screen of over 400 small molecules defined a set of multidrug resistance (MDR) genes for deletion strains sensitive to multiple drug treatments [30]. To distinguish between dhMotC-specific and more general cellular drug responses, we compared the 53 genes to the MDR gene list. None of the genes involved in the regulation of cellular pH were labelled as MDR genes, but 6 of 10 genes (60%) involved in vacuolar assembly and intracellular transport were. To further delineate the cellular response to dhMotC, we asked whether dhMotC directly affected vacuolar pH and intracellular transport.

### Effect of dhMotC on cellular pH

The chemical-genetic synthetic lethality screen revealed 10 of a total of 14 V-ATPase subunits (*CUP5*, *STV1*, *TFP1*, *TFP3*, *VMA2*, *VMA4*, *VMA6*, *VMA7*, *VMA10*, *VPH1*) and 2 membrane proteins required for its assembly (*VMA22*, *VPH2*) (Figure 6). This proton pump is a highly conserved multi-subunit enzyme complex that catalyzes the ATP-driven transport of protons from the cytoplasm to acidic organelles such as the vacuole and endosomes. As the central player in organelle acidification in all eukaryotic cells, the pump stores cellular energy in the form of a high concentration gradient of H^+ ^across organelle-delimiting membranes, thus constituting a large energy provider for the cell. Its proton motive force is implicated in a variety of cellular processes such as protein sorting in the biosynthetic and endocytic pathways, proteolytic activation of zymogen precursors, storage of metabolic building blocks, Ca^2+ ^homeostasis, and osmotic control [31].

**Figure 7:**
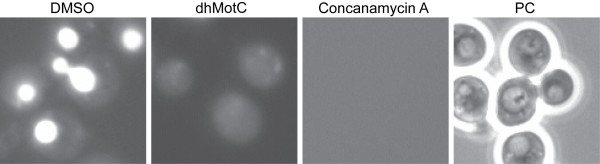
**DhMotC interferes with vacuolar acidification in yeast**. Quinacrine staining of yeast under different conditions: Cells were incubated with DMSO, 60 μM dhMotC or 50 μM concanamycin A, stained with the lysosomotropic dye quinacrine and visualized by fluorescence microscopy. Right panel shows control cells in phase contrast microscopy (PC).

In yeast, cellular pH can be assessed with the lysosomotropic amine quinacrine, a basic fluorescent compound that accumulates in acidified intracellular compartments such as the vacuole [32]. We used a quinacrine uptake assay to monitor the pH of vacuoles in dhMotC-treated yeast. As expected, non-treated cells accumulated quinacrine in the vacuoles, illustrating the acidic nature of the organelle (Figure 7). However, in cells treated with 60 μM dhMotC, quinacrine staining of the vacuoles could not be detected, indicating interference of the drug with the V-ATPase. A similar effect was observed with the specific V-ATPase inhibitor concanamycin A (Figure 7). The results suggest that dhMotC interferes with vacuolar acidification through the V-ATPase.

**Figure 8:**
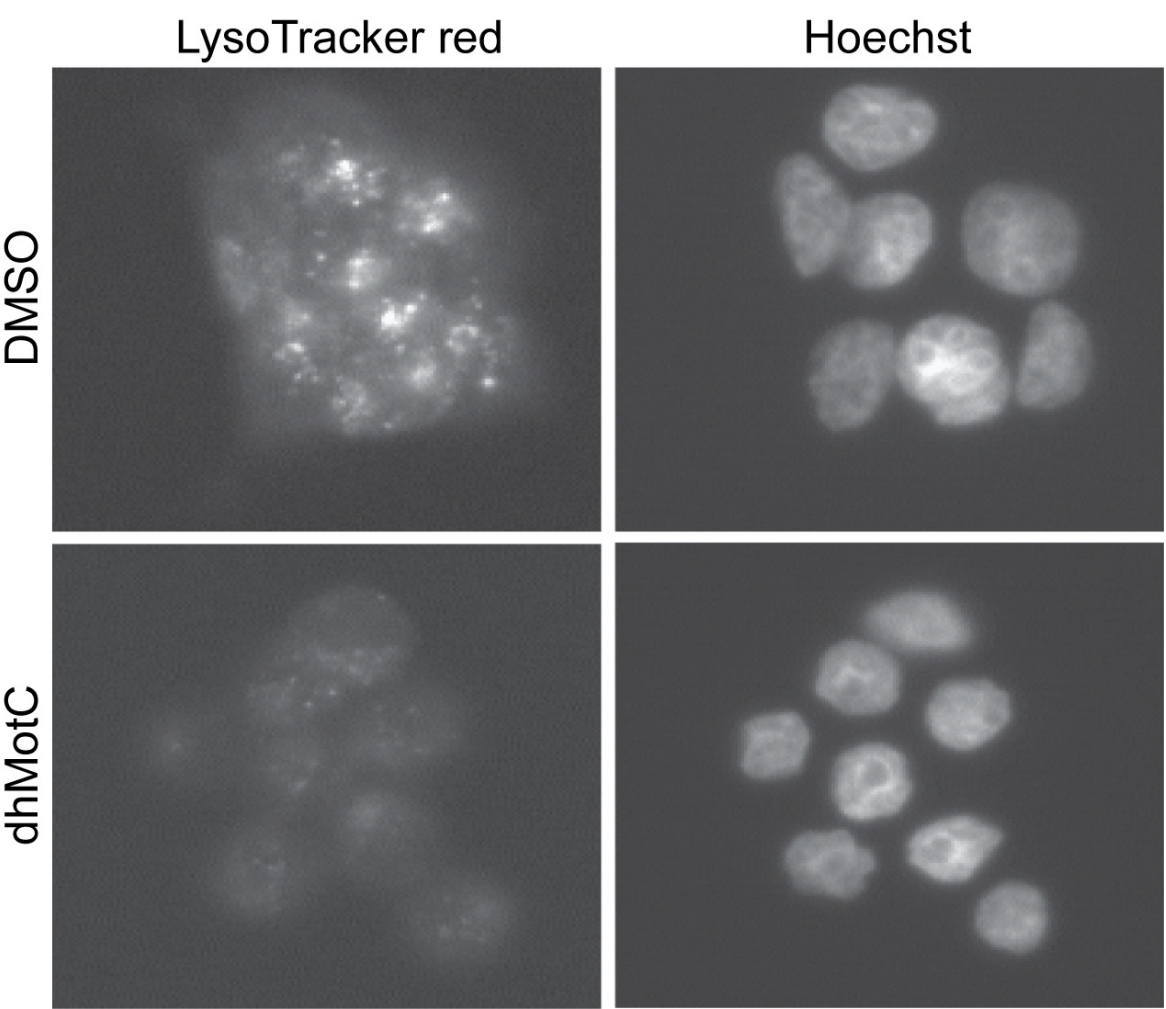
**DhMotC interferes with lysosomal acidification in cancer cells**. Cells were incubated with LysoTracker red followed by DMSO or 5 μM dhMotC, fixed and visualized by fluorescence microscopy. Right panels show nuclear stain.

We next examined whether dhMotC also affects the acidification of lysosomes in cancer cells. Human MDA-MB-231 breast carcinoma cells were incubated with LysoTracker red, a fixable fluorescent dye that accumulates in acidified compartments, treated with DMSO or dhMotC, fixed and examined by fluorescence microscopy. DhMotC caused a significant decrease in cytoplasmic LysoTracker red fluorescence intensity compared to DMSO-treated controls (Figure 8). Therefore, dhMotC interferes with lysosomal acidification in human cells as well as in yeast.

### Effect of dhMotC on vesicle-mediated transport

To gain additional insight into the involvement of the V-ATPase in the cellular effect of dhMotC and to confirm the results from the synthetic-genetic lethality screen, we monitored intracellular trafficking in drug-treated cells. Because a link between the V-ATPase, endocytosis and other transport mechanisms has been shown in several studies [31,33], we applied well-established assays to monitor endocytosis in yeast.

**Figure 9:**
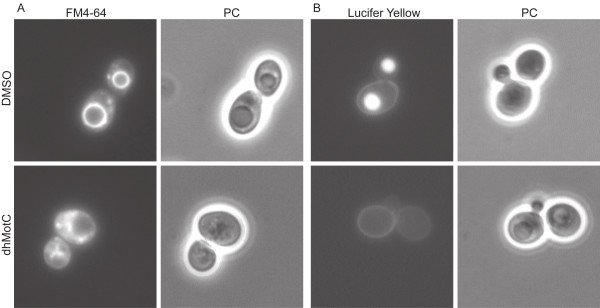
**DhMotC interferes with endocytosis in yeast**. Cells exposed to (A) FM4-64, a fluorescent endocytic marker staining the vacuolar membrane; (B) Lucifer yellow (LY), a fluid-phase endocytic marker accumulating in the vacuole. Cells were incubated with FM4-64 or LY in the presence of DMSO or 60 μM dhMotC and visualized after 90 min chase by fluorescence and phase contrast (PC) microscopy.

First, we followed membrane internalization and vesicle-based transport to the vacuole using FM4-64, a lipophilic styryl dye that incorporates into the cell membrane, is internalized and reaches the vacuole through an energy- and temperature-dependent transport mechanism. After 90 min in non-treated wild-type yeast cells, FM4-64 was entirely internalized and labelled the limiting vacuolar membrane (Figure 9A). Yeast cells treated with 60 μM dhMotC for 90 min were deficient in vesicle transport to the vacuole, as shown by residual fluorescent staining at the cellular membrane and accumulation of FM4-64 in small cytoplasmic vesicles (Figure 9A).

In a second assay, we monitored the delivery of Lucifer yellow (LY), a marker for fluid-phase endocytosis that accumulates in the vacuolar lumen. LY cannot cross biological membranes and, as a consequence, accumulation in the vacuole depends on vesicular transport. Untreated yeast cells displayed bright fluorescent staining of the vacuole by accumulated LY, whereas after 30 min of treatment with 60 μM dhMotC, LY failed to enter the cells and could only be detected as weak staining at the plasma membrane (Figure 9B). The results from the FM4-64 and LY assays confirm that dhMotC interferes with endocytosis.

As mentioned, killing of yeast by dhMotC depends on the presence of functional mitochondria. To test whether the disruption of endocytosis in drug-treated yeast cells was also mitochondria-dependent, we used the FM4-64 assay to monitor endocytosis in *ρ*^0 ^*petite *mutants. We observed a disruptive effect of dhMotC on endocytosis in both *ρ*^+ ^and *ρ*^0 ^cells (data not shown). Based on these results we concluded that, unlike death induced by dhMotC, inhibition of endocytosis did not require functional mitochondria.

We next examined whether motuporamines also inhibit intracellular membrane trafficking in cancer cells by examining effects on the internalization and degradation of epidermal growth factor (EGF) and its receptor (EGFR). Binding of EGF to EGFR at the plasma membrane leads to dimerization of EGFR, stimulation of its tyrosine kinase activity and initiation of downstream signaling cascades. The ligand-receptor complex is then downregulated via endocytosis and intracellular delivery to lysosomes for degradation [34].

**Figure 10:**
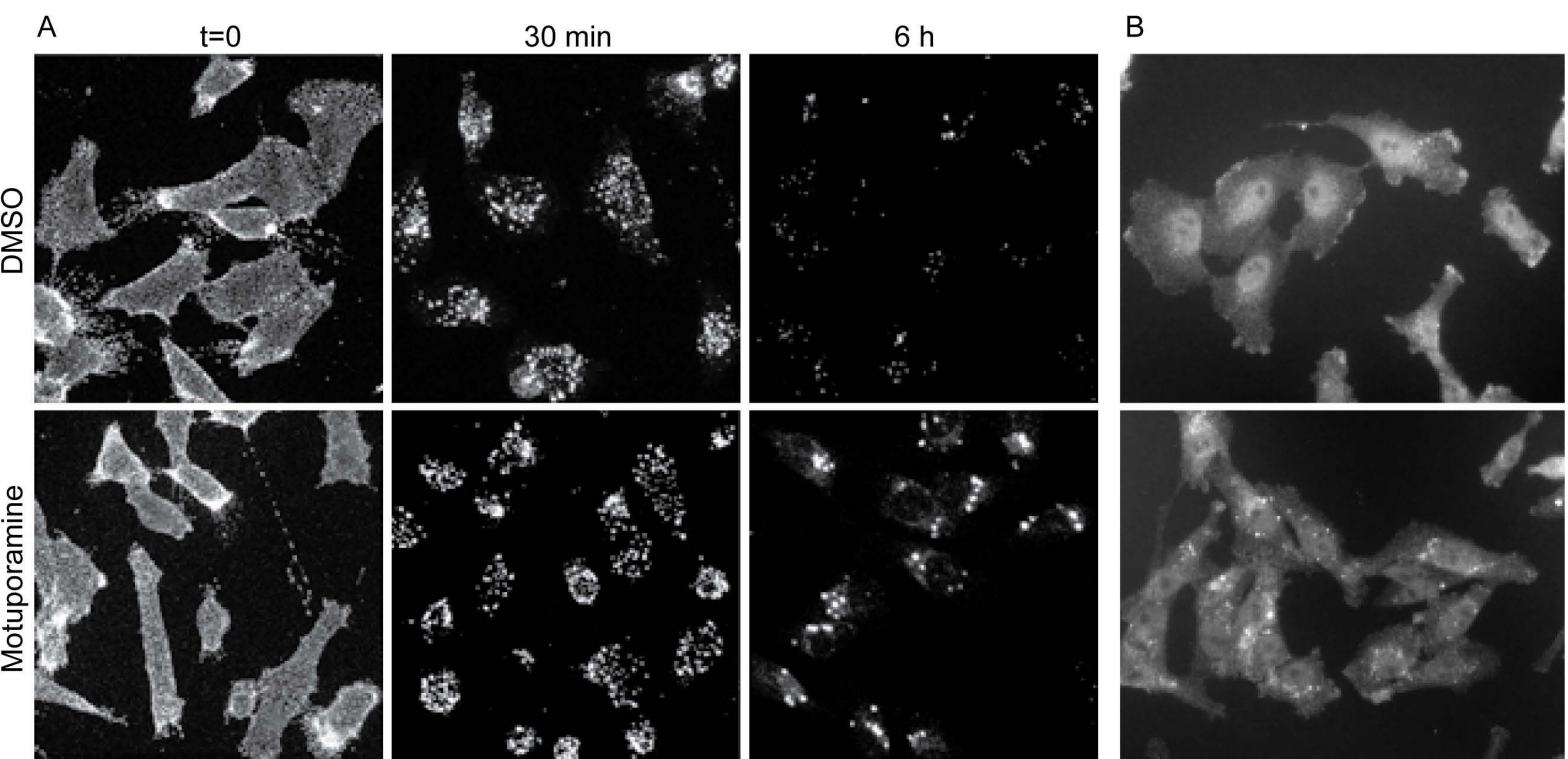
**Motuporamines inhibit the degradation of internalized FITC-EGF and causes intracellular accumulation of EGFR**. (A) Cells labelled with FITC-EGF at 4°C were exposed to DMSO (control) or 5 μM analogue 20 (motuporamine) for 0, 30 min or 6 h at 37°C, and FITC-EGF was visualized by fluorescence microscopy. (B) Cells were exposed to DMSO (control) or 5 μM analogue 20 for 24 h at 37°C, and EGFR was visualized by immunofluorescence microscopy.

MDA-MB-231 cells were incubated with fluorescently labelled EGF (FITC-EGF) for 1 h at 4°C, to enable binding of the ligand to its cell surface receptor. Cells were then incubated with DMSO or 5 μM motuporamine analogue 20 at 37°C for different times and examined by fluorescence microscopy (Figure 10A). At 4°C, FITC-EGF was bound to the cell surface. In both DMSO- and analogue 20-treated cells, EGF was internalized and showed a similar intracellular distribution for up to 1 h, indicating that the compound does not inhibit endocytosis or protein transport in the early endocytic pathway. After > 3 h, most of internalized FITC-EGF had disappeared from cells treated with DMSO, indicating it was degraded in lysosomes (Figure 10A). In contrast, cells treated with analogue 20 showed significantly more cytoplasmic punctate FITC-EGF, indicating that the compound interferes with the lysosomal delivery and/or degradation of internalized EGF.

To examine the effect of the compound on EGFR localization, cells were exposed to DMSO or dhMotC and the localization of EGFR was determined by immunofluorescence microscopy. In control cells, EGFR was present at the plasma membrane, with a noticeable concentration at the leading edge of migrating cells, as well as in intracellular structures (Figure 10B). In cells treated with dhMotC, EGFR was present in intracellular punctate structures and there was a clear reduction in plasma membrane-associated EGFR (Figure 10B), indicating that the compound interfered with the lysosomal delivery and/or degradation of internalized EGFR.

## Conclusion

A first screen of differential sensitivity of *ρ*^+ ^and *ρ*^0 ^cells showed that most drugs, including the therapeutic azole antifungals, do not require mitochondrial function to exert their growth inhibitory effects. Since *ρ*^0 ^cells appear incapable of generating ROS [35-38], ROS production by mitochondria is probably not a primary determinant of the mechanism of action of most antifungal agents. Only 4 chemicals required functional mitochondria to inhibit yeast growth. Antimycin A inhibits the transfer of electrons from ubiquinol to the cytochrome bc(1) complex. This inhibition is well known to cause the leakage of electrons to oxygen, resulting in the release of ROS [39]. Therefore, the inability of antimycin A to inhibit growth in *ρ*^0 ^cells can be attributed to the lack of ROS production due to the absence of a respiratory chain. Unexpectedly, *ρ*^0 ^cells were also resistant to 3 chemicals that target sphingosine and ceramide synthesis. Using dhMotC as an example, we showed that yeast cell killing requires holocytochrome c synthase activity. Moreover, forcing cells to rely exclusively on mitochondrial respiration to generate ATP by growth on a nonfermentable carbon source, rendered cells hypersensitive to dhMotC, highlighting the importance of a functional mitochondrial respiratory chain activity in its mechanism of action. These results indicate that sphingosine/ceramide biosynthesis is required to prevent mitochondria from becoming toxic to cells. In support of this conclusion, it has recently been shown that ceramide-depleted mitochondria were more sensitive to hydrogen peroxide and ethidium bromide [40] and that ceramide depletion in yeast mitochondria is associated with programmed cell death and oxidative stress [41].

A previous study from our laboratory explored drug-induced haploinsufficiency as a genome-wide approach to study the mechanism of action of drugs [6]. This work identified sphingosine/ceramide biosynthesis as the vital pathway inhibited by dhMotC. Interestingly, none of the 21 heterozygous mutants showing increased sensitivity to dhMotC was deleted of a gene involved in mitochondrial function. Therefore, the drug-induced haploinsufficiency screen, despite its genome-wide coverage, only partially revealed the mechanism of action of dhMotC, concealing genes of mitochondrial function involved in the mechanism by which dhMotC kills cells.

A second screen carried out in the present study, to identify suppressors of drug sensitivity, clearly showed that increasing the expression of genes encoding mitochondrial proteins can substantially increase resistance to dhMotC, further strengthening the link between mitochondria and the mechanism of action of the compound. Interestingly, comparing the results from the drug-induced haploinsufficiency screen [6] and the suppressor screen showed only 1 common gene, *SUI2*, a subunit of the translation initiation factor eIF2 involved in amino acid starvation [42]. This seemed surprising since the screens are conceptually similar in that they both rely on gene dose to identify drug-gene interactions. Differences between screens may be related to 1) stoichiometry, e.g. knockdown of 1 subunit of a protein complex is sufficient to reduce its activity and increase drug sensitivity while overexpression of 1 subunit of a protein complex is not sufficient to increase its activity and confer resistance, 2) redundancy, i.e. overexpression of a single gene is sufficient to confer resistance while knockout of redundant genes is necessary to detect sensitivity, and 3) unanticipated technical differences. Alternatively, the results may indicate a more complex relationship between gene dosage and drug sensitivity than has been generally considered.

The third screen carried out in this study was a chemical-genetic synthetic lethality screen to identify nonessential genes that increase sensitivity to dhMotC when completely deleted in haploid strains. 20 of the 53 genes identified have been implicated in resistance to multiple drugs in previous studies [30,43] and were therefore unlikely to provide clues about the specific mechanism of action of the compound. Standing out among the remaining genes are a number involved in the regulation of vacuolar pH, including 10 of 14 V-ATPase subunits and 2 membrane proteins required for V-ATPase assembly. This set of data strongly implicated vacuolar pH in the mechanism of action of dhMotC and led to the demonstration that dhMotC prevents vacuolar acidification. This effect is likely a consequence of inhibition of sphingosine/ceramide synthesis by dhMotC, since sphingolipids containing long-chain fatty acids are known to be necessary for V-ATPase activity [44].

Chemical-genetic synthetic lethality also revealed a large number of genes involved in vacuolar assembly and intracellular transport. Further experiments showed that dhMotC indeed inhibits the delivery of internalized FM4-64 to the vacuole as well as fluid phase endocytosis. This effect is also likely a downstream consequence of inhibition of sphingolipid synthesis since sphingolipids are important for protein trafficking [45] and endocytosis is blocked upon interruption of *de novo *sphingolipid biosynthesis [46]. Defects in vacuolar acidification and endocytosis caused by dhMotC occur in *ρ*^0 ^cells and are therefore independent of effects on mitochondria.

Interestingly, motuporamines also inhibited lysosome acidification and intracellular trafficking after endocytosis in cancer cells, demonstrating the capacity of this approach to predict targets in human cells. These results also provide insight into the mechanism by which dhMotC inhibits cancer cell invasion. EGF signaling plays an important role in cell migration [47]. Stimulation of cultured cancer cells with EGF increases invasion and motility and modulates cell adhesion to extracellular matrix components *in vitro *[48] and *in vivo *[49]. Overexpression of EGFR causes increased intravasation and lung metastasis from tumors implanted in the mammary fat pad, and cells overexpressing EGFR are more motile *in vivo *than adjacent cells not overexpressing EGFR [50]. By interfering with vesicle-mediated trafficking of EGFR, motuporamines considerably reduce plasma membrane-associated EGFR, and consequently its ability to control cancer cell migration.

In summary, this study demonstrates the value of using chemical genomics approaches in *Saccharomyces cerevisiae *to understand the mechanism of action of biologically active chemicals that may have human therapeutic value. However, reliance on a single genome-wide approach may often provide an incomplete picture of the mechanism of action of drugs. Different chemical genomics screens can provide complementary information and their combined use is probably necessary to provide a comprehensive understanding of the spectrum of different cellular effects a drug can have on cells.

## Methods

### Yeast strains, plasmids and growth conditions

The haploid set of viable yeast deletion mutants (mat_alpha_041902) was purchased from Invitrogen. The entire strain collection was used for the chemical-genetic synthetic lethality screen and individual strains (*aifΔ*, *mca1Δ*) were picked for drug sensitivity assays. Non-transformed yeast strains were grown in YPD (1% [wt/vol] yeast extract, 2% [wt/vol] bactopeptone and 2% [wt/vol] glucose), or YPgly (2% [vol/vol] glycerol) for media containing a nonfermentable carbon source. Respiratory-deficient *ρ*^0 ^strains were generated by inoculating 1 ml synthetic complete dextrose (SCD) medium (0.67% [wt/vol] yeast nitrogen base without amino acids, 2% [wt/vol] glucose, supplemented with appropriate amino acids) with 10 μl overnight yeast culture (BY4741 or FY1679-28C/TDEC) in the presence of 25 μg/ml filter-sterilized ethidium bromide. After 24 h incubation at 30°C and shaking at 200 rpm, 10 μl of the culture were transferred to 1 ml fresh ethidium bromide-containing SCD medium. After another 24 h shaking at 30°C, 100 μl culture was plated on YPD agar plates and incubated at 30°C for 2–3 days. For overexpression of *AVO1*, *ATP19*, *SDS22 *and *ACP1*, *S. cerevisiae *FY1679-28C/TDEC cells were transformed with *GAL1*-promoter driven BG1805 containing gene-specific open reading frames (ORFs). Plasmids were purchased as bacterial stocks from Open Biosystems. Transformed cells were grown in synthetic dropout-GAL medium (0.67% [wt/vol] yeast nitrogen base without amino acids, 1% [wt/vol] galactose and 1% [wt/vol] raffinose) supplemented with appropriate amino acids. For overexpression of mammalian Bcl-2, FY1679-28C/TDEC was transformed with a *GAL1*-driven pYES-DEST52 containing full-length human Bcl-2. Bcl-2 was purchased as an Ultimate™ ORF Clone from Invitrogen and the insert was transferred to the yeast expression vector through site-specific recombination (Gateway^® ^recombinases, Invitrogen).

Compounds were obtained from the Canadian Chemical Biology Network Chemical Collection sourced from Prestwick, Biomol, Sigma and Microsource. Motuporamines were a generous gift of D. Williams (University of British Columbia). They were synthesized as described [51] and solubilised in DMSO. Myriocin and suloctidil were purchased from Sigma and solubilised in DMSO. Quinacrine dihydrochloride and Lucifer yellow CH were purchased from Sigma and solubilised in H_2_O or medium. FM4-64 was purchased from Invitrogen.

### Halo toxicity screen

A solution of YPD with 2% agar was prepared by dissolving 5 g of yeast extract, 10 g of peptone and 10 g of agar in 450 ml H_2_O. After autoclaving and cooling to 65°C, 50 ml of filter-sterilized 20% glucose solution was added. 45 ml of medium were dispensed in Omnitray plates and left to set. A solution of YPD with 0.5% agar was prepared the same way by adding 2.5 g agar. For each plate screened, 23 ml YPD 0.5% agar were inoculated at 50–55°C with 500 μl of an overnight yeast culture (FY1679-28C/TDEC, BY4741 or *ρ*^0 ^mutants of the same strains) and 22 ml of the mixture were poured in the Omnitray plates on top of the set YPD 2% agar and left to set for 1 h.

Compounds were pin-transferred from 5 mM DMSO stocks in 96-well plates into the cooled agar with a pin-tool robot (BG600, BioRobotics) using 0.7 mm dia. pins that deliver 0.34 μl each. Before and between applications pins were cleaned by submersion in 10% bleach and 70% ethanol for 5 s each followed by drying for 10 s with warm sterile air. The plates were incubated at 30°C for 48 h and halos were verified by visual inspection.

### Growth inhibition measurement in liquid culture

Yeast strains (OD_600 _= 0.02) were incubated with appropriate dilutions of each compound in 200 μl cultures in 96-well plates, in addition to DMSO controls. Kinetic growth curves were generated with a TECAN plate reader by reading the OD every 2 h after agitating the plate prior to reading to suspend the yeast. For growth comparisons between different treatments the exponential part of the growth curve was considered and ODs were transformed into log_10 _values. The least squares method was applied to generate a straight line that best fit the data and line slopes were calculated to compare growth behaviour between different growth conditions.

### Drug dosage suppressor screen

Multicopy pool construction and growth – A *S. cerevisiae *random genomic library (gift from Martha Cyert) constructed in a high-copy 2 micron expression vector (YEplac195) with an average insert size of approximately 5 kb was transformed into yeast (BY4743) by a standard lithium acetate method [52] and selected in -URA dropout medium. After 3 days incubation at 30°C, ~106 transformants were pooled into medium containing 7% DMSO, aliquoted, and stored at -80°C. For screens, frozen aliquots were thawed and inoculated directly into 700 μl -URA dropout medium to an OD_600 _= 0.02. Compound was added and the pool was grown for 5 generations in 48-well microtiter plates (Nunc). Final compound concentrations were as follows: 50 μM for dhMotC, analogue 20 and 27, 6 μM for analogue 21. An inhibitory concentration of at least 50% (IC_50_) was necessary to provide sufficient selection when screens were performed for 5 generations. Cells were harvested automatically by a Packard Multiprobe II four-probe liquid-handling system (PerkinElmer). Plasmid isolation, insert PCR amplification and microarray hybridization – Plasmids were isolated using the Zymoprep II plasmid isolation kit (Zymo Research). The inserts were amplified by PCR with the FailSafe™ PCR System (Epicentre^® ^Biotechnologies) using common M13 primers. PCR cycling conditions were: an initial melting step at 95°C for 2 min followed by 30 cycles at 95°C for 0.5 min, 58°C for 0.5 min and 68°C for 10 min followed by a final extension at 68°C for 15 min. The PCR products were purified using the QIAquick PCR purification kit (Qiagen) and labelled with biotin using the BioPrime labelling kit (Invitrogen). Labelled products were hybridized to Affymetrix TAG4 arrays using the same protocols as described for TAG hybridizations [53]. Multicopy suppression profiling (MSP) analysis – ORF probe intensities were extracted and normalized. Log2 ratios of probes for drug-treated versus DMSO control were calculated. Each ORF was represented by at least 2 probes and the log2 ratios were averaged to generate a single score for each gene. To identify each suppressor locus, the log2 ratios of intensities were ordered by each ORF's genomic location and analyzed using a sliding window to identify loci that had at least 2 adjacent ORFs with log2 ratios ≥ 1.6.

### Quinacrine assay

Wild type yeast (BY4741) was grown overnight in YPD buffered with 50 mM NaH_2_PO_4 _at pH 7.6. Cells were harvested by centrifugation (1 min, 13000 rpm, RT, Hereaus pico microcentrifuge) and resuspended in 200 μl phosphate-buffered YPD at OD_600 _= 0.3. Compounds were added and yeast was preincubated for 1 h in the presence of 60 μM dhMotC or 100 μM concanamycin A. For labelling with quinacrine, 4 μl of 10 mM stock were added to a final concentration of 200 μM and the mixture was incubated at RT for 5 min. Cells were harvested by centrifugation and washed with SCD medium buffered at pH = 7.6. For visualization yeast cells were resuspended in 10–20 μl buffered YPD.

### Yeast endocytosis assays

For the FM4-64 assay, yeast cells were grown overnight and the cell count was adjusted to OD_600 _= 1.2. Cells were divided in 200 μl aliquots and cells were preincubated at 30°C in the presence of 60 μM dhMotC or DMSO. Cells were harvested by centrifugation and resuspended in 10 μl YPD. 2 μl of FM4-64 diluted 100 × were added and the mixture was incubated on ice for 30 min. After harvesting and washing with H_2_O, cells were resuspended in 20 μl YPD in the presence of 60 μM dhMotC or DMSO and incubated at 30°C for 1 1/2 h. To terminate the assay, 1 ml of ice-cold 50 mM potassium phosphate buffer containing 10 mM NaF and 10 mM NaN_3 _was added. For visualization, yeast cells were harvested and resuspended in 20 μl potassium phosphate buffer. For the Lucifer yellow assay yeast cells were grown to OD_600 _= 0.1. After harvesting by centrifugation the pellet of yeast cells was resuspended in 90 μl YPD medium and 10 μl of 40 mg/ml Lucifer yellow stock was added to a final concentration of 4 mg/ml. DhMotC was added immediately to a final concentration of 60 μM. The mixture was incubated at 30°C with shaking at 200 rpm for 1 1/2 h. To stop the assay, 1 ml of ice-cold 50 mM potassium phosphate buffer containing 10 mM NaF and 10 mM NaN_3 _was added. Cells were harvested and washed 3 × with 1 ml ice-cold potassium buffer. After the last wash, cells were resuspended in 20 μl buffer for visualization. A Zeiss microscope (Axiovert S100) equipped with filters for epifluorescence and phase contrast was used. Cells stained with quinacrine or Lucifer yellow were observed by exciting with 420–490 nm light and viewing emitted light with a 520–550 nm filter. Cells stained with FM4-64 were observed by exciting with 520–550 nm light and viewing emitted light with a 610 nm cut-off filter. Photographs were taken with a QImaging Microimager II camera. Data were collected as pictures of random fields and each field was photographed both with phase contrast optics and epifluorescence.

### Cancer cell assays

MDA-MB-231 cells were grown in DMEM/F12 supplemented with 5% fetal bovine serum and 5 μg/ml insulin. For the LysoTracker red assay, cells grown on coverslips were incubated with 100 nM LysoTracker red (Molecular Probes) for 25 min before addition of chemicals for 35 min. Cells were fixed with 3.7% paraformaldehyde in PBS, washed and DNA was stained with Hoechst 33342. For EGF internalization assays, cells grown on coverslips were incubated at 4°C for 1 h with 0.4 μg/ml FITC-EGF (Molecular Probes) in cell culture medium supplemented with 2 mg/ml bovine serum albumin. Cells were then washed twice with cold medium before adding chemicals in cell culture medium at 37°C. After different times at 37°C, cells were fixed with 3.7% paraformaldehyde in PBS, washed twice and mounted on slides for microscopy. For EGFR immunostaining, cells grown on coverslips were fixed with 3.7% paraformaldehyde in PBS, permeabilized with 0.6% Triton X-100 in PBS, blocked with PBS containing 10% fetal bovine serum and 2% bovine serum albumin, incubated with 3 μg/ml monoclonal anti-EGFR antibody (Merck), washed and further incubated with CY3-conjugated goat anti-mouse IgG, F(ab') fragment-specific antibody (Jackson Laboratory).

## Authors' contributions

DK, LMM, DR, GG, CN, CDR and MR conceived and designed the experiments. DK, LMM, SH and DR performed the experiments. DK and LMM analyzed the data. DK and MR wrote the paper. All authors read and approved the final manuscript.
